# p53 Isoforms as Modifiers of the p53-Dependent Responses: A Hidden Code?

**DOI:** 10.3390/cancers18071057

**Published:** 2026-03-25

**Authors:** Laura Bartolomei, Beatrice Pretto, Samuele Brugnara, Alessandra Sontacchi, Vanessa Dassi, Aya Bousrih, Chiara Damaggio, Francesca Flangini, Alessandra Bisio, Yari Ciribilli

**Affiliations:** 1 Laboratory of Radiobiology, Department of Cellular, Computational and Integrative Biology (CIBIO), University of Trento, Via Sommarive 9, 38123 Povo, TN, Italy; laura.bartolomei@unitn.it (L.B.); beatrice.pretto@studenti.unitn.it (B.P.); alessandra.sontacchi@unitn.it (A.S.); francesca.flangini@studenti.unitn.it (F.F.); 2 Laboratory of Molecular Cancer Genetics, Department of Cellular, Computational and Integrative Biology (CIBIO), University of Trento, Via Sommarive 9, 38123 Povo, TN, Italy; samuele.brugnara@unitn.it (S.B.); vanessa.dassi@studenti.unitn.it (V.D.); bousrih.aya97@gmail.com (A.B.); chiara.damaggio@studenti.unitn.it (C.D.); 3 Trento Institute for Fundamental Physics and Applications (TIFPA), Istituto Nazionale di Fisica Nucleare (INFN), Via Sommarive 14, 38123 Povo, TN, Italy

**Keywords:** p53 isoforms, *TP53*, cancer biomarkers, cancer aggressiveness, gene expression

## Abstract

The tumor suppressor protein p53, encoded by the *TP53* gene, is known as the “Guardian of the Genome”, and alterations in *TP53* are common to more than 50% of human cancers. p53 is a critical regulator of cellular responses to several stress conditions, such as DNA damage, oncogene activation, and nutrient starvation. p53 was traditionally described as a single transcription factor; however, now it is recognized as a complex family of isoforms generated through alternative promoter usage, alternative splicing, and alternative initiation of translation. These processes give rise to at least 12 distinct p53 isoforms in humans, including p53α (the canonical full-length isoform), p53β, p53γ, Δ40p53, Δ133p53, and Δ160p53, each with unique structural and functional properties. p53 isoforms differ in the presence or absence of specific and fundamental domains located both at N- and C-terminal ends, determining an altered DNA-binding potential, transcriptional activity, and protein–protein interactions. For instance, Δ133p53 isoforms lack part of the N-terminal domains and can exert dominant-negative effects over full-length p53α or modulate alternative transcriptional programs. Similarly, p53β and p53γ isoforms, which have a unique C-termini, influence cellular senescence. The expression patterns of p53 isoforms are tissue-specific and dynamically regulated under both physiological as well as pathological conditions. Alterations of isoform balance have been involved in tumor progression, metastasis, and therapy resistance. Importantly, specific isoforms can either enhance or limit canonical p53 tumor suppressor functions, thereby contributing to the functional diversity of the p53 network. Overall, the p53 isoform landscape adds a critical layer of complexity to p53 biology. In this review, we summarize the mechanisms underlying the production of p53 isoforms, their functions, and their expression in cancer, with the idea that a better understanding of the differential regulation and functional interplay of p53 isoforms may provide novel biomarkers and therapeutic targets in cancer.

## Simple Summary

The tumor suppressor protein p53 is known as the “Guardian of the Genome” and protects our body from cancer development. Indeed, alterations in p53 are common to more than half of all human cancers. In response to stressful conditions such as DNA damage, uncontrolled growth signals, or lack of nutrients, p53 gets activated and induces growth arrest or cell death. Scientists once believed that p53 was a single protein, but it is now accepted to exist in at least twelve different forms, named p53 isoforms. These slightly different variants can be produced naturally and exhibit multiple behaviors. Some of these isoforms can support or even potentiate canonical p53 functions, while others can weaken or modify it. Importantly, the balance between p53 isoforms varies across tissues. When this balance is disrupted, it can contribute to cancer aggressiveness, metastasis, and resistance to therapy. In this review, we summarize the mechanisms behind the production of p53 isoforms and their relevance in cancer.

## 1. Introduction

*TP53* is a tumor suppressor gene involved in genome stability maintenance and cancer formation prevention. It retains a crucial role in the complex signaling cascade activated in response to cellular damage, represented by the recognition and repair of DNA lesions. The proper modulation of gene expression by p53 transcription factor is involved in cell fate decision, particularly between cell cycle arrest and apoptosis. The importance of p53 is demonstrated by the fact that its tumor-suppressive activity is ubiquitously lost in almost 50% of human cancers as a result of p53 protein inactivation or *TP53* gene mutation [1,2]. Interestingly, and contrarily to most tumor suppressor genes, the vast majority of *TP53* alterations involves missense mutations, while nonsense mutations are rarer, and deletions or frameshift mutations are very uncommon [3]. The reason for this characteristic relies on the ability of the hotspot p53 mutations (mainly localized in the DBD, see below) to acquire gain-of-function ability (stimulate proliferation, support angiogenesis, induce cancer aggressiveness and favor metastasis) and dominant-negative potential in the heterozygous condition [4,5]. The *TP53* gene is located on the short arm of chromosome 17 (17p13) and spans about 20kb. It is composed of 10 introns and 11 exons, the first of which is non-coding [6]. It encodes the human p53 protein (393 amino acids) which is subdivided into six functional domains: a transactivation domain (TAD), needed for the transcriptional activation of p53 target genes and composed of two complementary transcriptional activation domains (TAD1 critical for acute DNA damage responses, TAD2 crucial for p53-dependent apoptosis and both involved in the regulation of p53 tumor suppressor functions) [7,8]; a proline-rich domain (PRD), mostly involved in protein–protein interaction, transcriptional repression, reactive oxygen species (ROS) production and apoptosis modulation [9,10]; a DNA-binding domain (DBD) that regulates p53 interactions with target chromatin regions; three nuclear localization signals; an oligomerization domain (OD) that mediates the formation of the p53 tetramer (the regulation of p53 tetramerization affect DNA binding activity, protein–protein interactions, post-translational modifications and p53 degradation); and a C-terminal regulatory domain, the site of multiple post-translational modifications that negatively modulate the p53-DNA binding activity.

The binding of wild-type p53 to the DNA is directed by the presence of p53 response elements (REs) in the regulatory regions of p53-regulated genes. The p53 RE has a consensus sequence defined by two copies of a degenerated 10-bp motif, 5′-RRRCWWGYYY-3′, where R stands for purines, W for A/T and Y for pyrimidines, with a spacer of up to 13 bp that provides for the binding of a p53 tetramer [11]. Through DNA binding, p53 controls and modulates the transcription of target genes involved in cell cycle control, apoptosis, senescence, metabolism, differentiation, and DNA repair, as well as p53 own turnover and modulation. Depending on the selectivity and kinetics of gene activation and repression, different processes are activated [12]. The fine tuning and balance between p53 transcription and degradation is fundamental, since excessively low levels of p53 can allow cancer development, whereas too high levels can be lethal to cells [13].

p53 is rapidly stabilized and accumulated in response to alteration of normal cell homeostasis, represented mainly by DNA damage, nutrient starvation, heat shock, virus infection, pH change, ribosomal/nucleolar stress, hypoxia, and oncogene activation. Instead, in the absence of cellular stress, basal p53 protein levels are kept low by the activity of the ubiquitin ligases as HDM2 (known as MDM2, that stands for mouse double minute 2) and PIRH2 [14]. MDM2 negatively regulates p53 activity via two main processes. Firstly, it directly binds the N-terminal region of p53, inhibiting the transcriptional activation function of p53. Secondly, MDM2 is an E3-ubiquitin ligase that promotes p53 proteasomal degradation. In the nucleus, in a complex with the CBP (p300/CREB-Binding Protein, a transcriptional coactivator protein that serves as scaffolding), MDM2 catalyzes p53 mono-ubiquitination onto lysine residues in the C-terminus. p53 mono-ubiquitination represents a signal for nuclear export rather than a substrate for proteasome degradation [15]. The degradation is induced mainly thanks to the final p53 poly-ubiquitination that occurs into the cytosol mediated by p300/MDM2 complexes [16]. Upon oncogenic signaling or ribosomal/nucleolar stress, the binding of specific proteins—p14^ARF^ tumor suppressor protein, ribosomal proteins, and nucleolar proteins—to MDM2 inhibits its function and disrupts p53-MDM2 interaction, thereby promoting p53 stabilization and its function as a transcription factor [17,18]. In addition, post-translational modifications, predominantly phosphorylation, disrupt MDM2-p53 interaction, leading to p53-dependent effects on gene expression [18,19]. Active p53 protein regulates the transcription of target genes involved in cell cycle arrest, senescence, and programmed cell death. In the transition from G1 to S cell cycle phase, p53 mediates transient or irreversible cell cycle arrest via the transcriptional activation of p21, a cyclin-dependent kinase (CDK) inhibitor [20]. Indeed, p21 is required for the induction of G1 arrest. For the maintenance of G2 arrest, GADD45 and 14-3-3σ are also activated. They also regulate G2/M progression in response to ionizing radiation [21,22]. In response to cellular stress, molecules involved in both extrinsic and intrinsic apoptotic pathways are regulated by p53. In some cases, the induction of some of these genes is enough to initiate cell death, while the induction of other genes by themselves will not cause cells to undergo apoptosis [23].

Regarding all these processes, the main point is represented by the choice of response to p53 that drives cell fate between cell cycle arrest and apoptosis [13]. Several studies try to explain this suggesting that (i) p53 induces genes involved in these two biological processes to the same extent in all conditions, although additional and independent transcription factors intervene, or (ii) p53 itself is responsible for differential expression of apoptotic genes according to p53 cell levels or p53 post-translational modifications that affect its DNA binding or its interaction with transcriptional coactivators [24]. As mentioned, wild-type p53 has multiple roles in regulating cell growth, cell death, DNA repair, differentiation, development and more, and its alterations are key players in cancer development. Hence, it is reasonable to suggest that these multiple activities may be mediated by multiple p53 protein molecules. Indeed, p53 isoforms are currently acknowledged to regulate the p53 tumor-suppressive functions, and their dysregulation is found in many cancers [25,26]. p53 isoforms with their activity and levels represent an additional layer of complexity and regulation. They can exhibit both oncogenic and tumor suppressor functions, thus influencing the cell response to stresses like DNA damage [27,28,29]. p53 isoforms are, in fact, a recent topic of biological interest, since they are recognized as potential modifiers of p53-dependent responses [30].

The p53 gene family (p53/p63/p73) members have in fact a dual gene structure conserved in Drosophila, zebrafish, and humans. They express multiple mRNA variants due to alternative promoters and multiple splicing, leading to the generation of different forms of proteins containing different domains of them, called isoforms.

Several mRNAs can be transcribed from the *TP53* gene given that the gene transcription can be initiated from two distinct promoters: the canonical P1 promoter, located upstream of exon 1, or an internal P2 promoter, located within intron 4. Moreover, *TP53* mRNAs can be alternatively spliced at intron 2 or intron 9, generating variants with different N- and C-termini. Lastly, *TP53* mRNAs can also be translated starting from different codons, leading to the formation of isoforms of different length [30].

**Figure 1:**
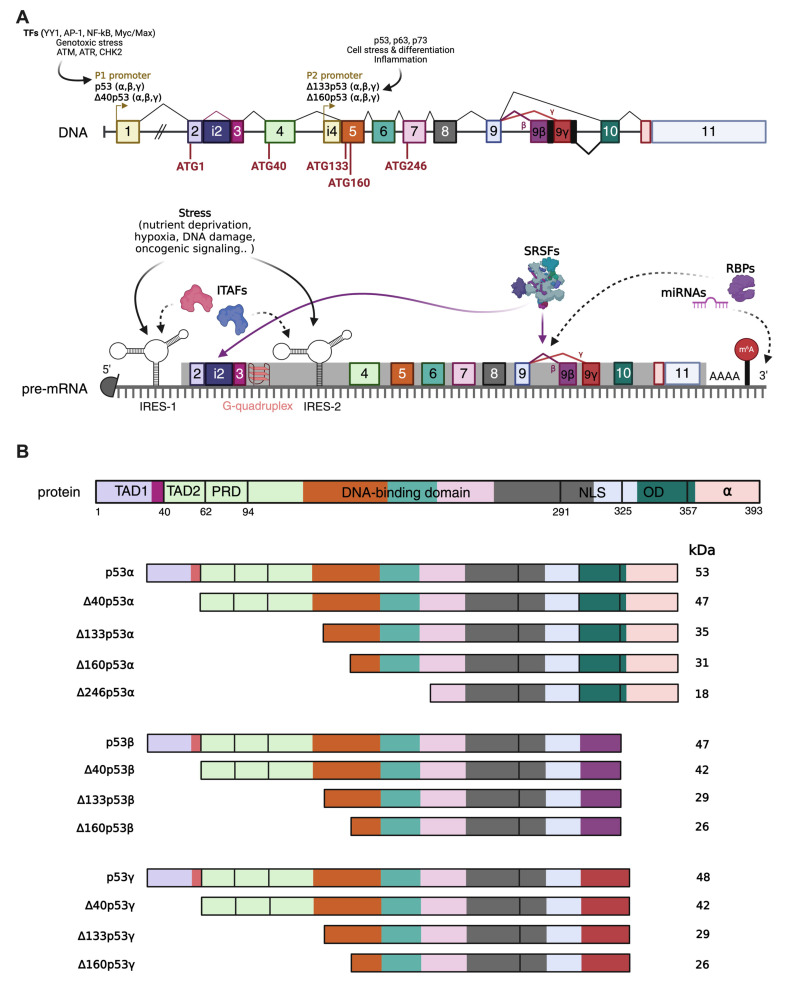
p53 isoforms’ structure and regulation. (**A**) A representative image of the *TP53* gene locus (upper panel) with introns and exons. P1 and P2 promoters and multiple ATG sites are indicated. Numbers indicate the different exons that can be distinguished by a color code, which is maintained in both panels to show the parts of the protein they encode. Sequence of the *TP53* pre-mRNA (lower panel) and its post-transcriptional regulation. Two Internal Ribosomal Entry Sites (IRES) can regulate translation initiation at the canonical 5′-UTR for full-length p53 forms and at codon 40, hence promoting Δ40p53 forms’ production. Translation initiation at these two sites is also favored by IRES Trans-Acting Factors (ITAFs). G-quadruplexes structures enhance alternative splicing at intron 2. In addition, serine/arginine-rich splicing factors (SRSFs) fine-tune alternative splicing and exon exclusion/inclusion by binding to specific cis-acting elements within the pre-mRNA. microRNAs (miRNAs), RNA-binding proteins (RBPs), and N6-methyladenosine (m6A) modifications may influence differential isoforms expression. (**B**) Protein structure organization of the full-length p53 (FLp53) and longer/shorter p53 isoforms. Functional domains of p53 are illustrated: transactivation domains (TADs), proline-rich domain (PRD), DNA-binding domain, three nuclear-localization signals (NLS), oligomerization domain (OD), and C-terminal regulatory region. Different colors represent the exons that compose the protein. Created with Biorender.com.

All these mechanisms allow the generation of at least 12 p53 isoforms (Figure 1). Longer isoforms are represented by full length, canonical p53 (FLp53) and Δ40p53 isoforms. Canonical p53 isoforms are transcribed from P1 promoter and translated starting from codon 1. Alternative splicing at intron 9 generates three different isoforms: canonical p53 (also called p53α), p53β, and p53γ of which p53β and p53γ lack the oligomerization domain with respect to canonical p53 [31]. Δ40p53 isoforms (α, β, γ) are translated starting from codon ATG40, leading to the lack of the TAD1 domain. Δ40p53 isoforms can also be translated from IRES (internal ribosomal entry sites) and controlled by IRES transactivating factors (ITAFs). Δ40p53 forms are also obtained due to alternative splicing of the intron 2. Instead, shorter isoforms include Δ133p53 (α, β, γ) and Δ160p53 (α, β, γ) [32]. They are transcribed from internal P2 promoter and translated respectively from codon 133 and codon 160 (Figure 1A). They lack TAD1, TAD2, PRD, and part of the DBD. Δ133p53 and Δ160p53β and γ isoforms are derived as a consequence of alternative splicing of intron 9 as for the longer p53 isoforms [30,33,34]. Interestingly, a shorter Δ160p53 variant starting nine codons downstream, Δ169p53, was described with very similar functions to Δ160p53 (i.e., favoring cancer cells’ survival) [35]. Recently, a new short Δ246p53α isoform has been discovered. It is translated from an alternative translation initiation site (TIS) at codon 246 within exon 7, and it is induced upon DNA damage in cancer and non-cancer cell lines, demonstrating a role in senescence induction and inhibition of tumor growth [36] (Figure 1B). Very recently, in a young rhabdomyosarcoma patient with a Li-Fraumeni-like family predisposition, the E224A *TP53* mutation was identified. This mutation determined the skipping of the exon 6 with the production of a smaller p53 variant [37]. Interestingly, this short p53 isoform was also detected in a cohort of Chronic Lymphocytic Leukemia (CLL) [38] and in breast/ovarian cancer [39].

The expression of p53 isoforms is not only regulated at transcriptional and translational levels. Epigenetic events can regulate the tissue specificity of the p53 promoters, and the internal *TP53* promoter activity is influenced by several polymorphisms, like the 16-bp insertion/duplication in intron 3 (rs17878362) and the R72P polymorphism in exon 4 (rs1042522), that alter Δ133p53 isoforms expression. The internal P2 promoter can be transactivated by p53α, p63, and p73 isoforms and p68. Therefore, cell type (epigenetically) defines the pattern of p53 isoforms expression and can influence the differential possibilities of cell-fate outcome.

The stability of the different isoforms in the cells is also regulated by p53 degradation pathways, such as the MDM2-mediated ubiquitination-26S proteasome pathway. p53 isoforms have been demonstrated to be biologically active either alone or in combination, and they differentially regulate gene expression. The balance of expression among them and their interaction with canonical p53α plays also a role in defining the p53-dependent cell response upon DNA damage, virus/bacterial infection or cellular signals [26]. Hence, p53 isoforms play a crucial role in cell cycle progression, programmed cell death, senescence, inflammation, stem-cell renewal and differentiation, aging, neurodegeneration, glucose metabolism, angiogenesis, embryo development and cancer [30]. The Δ40p53 isoform can form homo-tetramers and bind to the promoter of 14-3-3σ, a member of the 14-3-3 protein family that interacts with p53 and modulates its activity. This interaction activates 14-3-3σ expression and induces cell cycle arrest in response to endoplasmic reticulum (ER) stress. In addition, Δ40p53 as well as p53β expression can promote tumor cell senescence through the up-regulation of p21 [40,41].

In contrast, Δ133p53 has been shown to inhibit senescence by repressing p21 and miR-34a expression, thereby preventing canonical p53 from binding to the promoters of these genes [30,42]. Regarding apoptosis, p53β can form a complex with p53α, enhancing its transcriptional activity on the BAX promoter and thereby promoting apoptosis. When Δ40p53 expression is low or comparable to that of p53α, it similarly induces BAX expression. Conversely, Δ133p53 isoforms antagonize p53-mediated apoptosis by inducing the expression of anti-apoptotic members of the Bcl-2 family.

Overall, altered expression of p53 isoforms may impact cancer cells’ aggressiveness and response to therapy [26,43]. In this review we will summarize (a) the regulation of the endogenous levels of the different p53 isoforms and the known mechanisms underlying it and (b) how the differential expression of each p53 isoform can influence cancer development, aggressiveness, and prognosis.

## 2. Molecular Mechanisms for the Regulation of p53 Isoforms’ Expression

### 2.1. Promoter-Dependent Regulation of p53 and Its Isoforms

The expression of p53 and its isoforms is regulated at the transcriptional level by its dual gene structure, which contains two distinct promoters. Presently, *TP53* is known to produce twelve different isoforms, six of them produced from the canonical upstream promoter (P1), and six (or seven, as previously mentioned) of them produced starting from the internal promoter P2 [30].

Transcription from P1 gives rise to the full-length p53 (p53α) and, through alternative splicing, the C-terminal variants p53β and p53γ. Thanks to an internal ribosomal entry site (IRES) at codon 40 and alternative splicing in intron 2, the N-terminally truncated Δ40p53 isoforms are also derived from transcription initiation at P1 promoter [44]. On the other hand, transcription can also be initiated from the internal promoter P2 located within intron 4, leading to the production of Δ133p53, Δ160p53, and Δ246p53 isoforms, via the same mechanisms described for the previous isoforms [32,44].

Transcription starting from each of the two promoters is highly variable across cell types and cellular contexts; in particular, p53 transcriptional control plays a fundamental role in mitogenic stimulation, induced differentiation, and response to genotoxic stress. p53 is constitutively produced at low levels, with the P1 promoter that can be modulated by several transcription factors that bind to specific promoter motifs, such as Ying Yang 1 (YY1), Activating protein 1 (AP-1), Nuclear Factor-kappaB (NF-κB), and Myc/Max, which have been demonstrated to be required for an efficient transcription initiation. AP-1, NF-κB, and c-Myc cooperate for p53 transcriptional up-regulation in response to mitogenic stimulation. This effect is of physiological importance to ensure genomic stability maintenance in actively cycling and dividing cells [45,46]. The canonical promoter of p53 can also be regulated by p53 itself in an autoregulatory feedback loop, both in normal conditions and under DNA-damaging conditions. This effect seems to be cell type-dependent, as it has been shown that p53 can up- or down-regulate its own transcription depending on the cellular model [47,48,49]. Another way to regulate p53 transcription involves hormone signaling, with 17β-estradiol (E2)-activated Estrogen Receptor (ERα) or CCAAT-binding transcription factor-1 (CTF-1), and NF-κB can also bind and induce the production of p53 mRNAs [50,51].

Furthermore, the expression of p53 and its isoforms from the P1 promoter is increased following genotoxic stress, after the activation of signaling pathways involving ATM/ATR and CHK2, which phosphorylate and stabilize the transcription factor Che-1 [52].

Regarding the transcription of p53 isoforms from the P2 promoter, a principal regulator is p53 itself, which can positively modulate transcription by binding to a response element located upstream of the P2 transcription start site within intron 4 [53,54]. This induces the production of the shortest N-terminally truncated Δ133p53 and Δ160p53 isoforms. p53 is recruited to the P2 promoter in the presence of genotoxic stress, modulating p53 response to stress signals [55]. In addition, other members of the p53 family can influence the P2 promoter, as p63, p73, and their isoforms have been demonstrated to bind to the internal promoter in a cell type-dependent manner and in response to cell stress and differentiation [56]. P2 promoter-dependent transcription can also be influenced by inflammation pathways, such as in the case of infection by *H. pylori*, where Δ133p53 and Δ160p53 isoforms are induced by the action of the AP-1 complex [57].

Additionally, polymorphisms in the P2 promoter are another important characteristic influencing the production of p53 shorter isoforms. Different SNPs lead to differential activity of the promoter and variable levels of the isoforms [58], which affect the functionality of p53 and have also been linked with cancer progression [59]. Specifically, there are eight known SNPs that can have an impact on P2 promoter regulation. The most studied *TP53* polymorphism in this region is P72R polymorphism (rs1042522 G>C) within exon 3 (which is part of the regulatory elements of P2 promoter), where a G is changed in C altering the codon 72 from Proline to Arginine [60]. Additionally, two major SNPs in intron 4, rs9895829 (A>G) and rs2909430 (C>G), lie within the 5′-UTR of the p53 isoforms starting from promoter P2. These SNPs have been demonstrated to influence the production of p53 shorter isoforms Δ133p53 and Δ160p53 and thus can potentially play a role in cancer prognosis [58,59]. Noteworthily, even if on a limited number of samples, we have recently shown that the heterozygous condition in polymorphism rs1042522 (P/R) tended to be correlated with elevated Δ133/Δ160p53 (mainly β and γ) in uveal melanoma patients when compared with homozygous P72R [43].

Overall, promoter P2 acts as a stress- and context-responsive promoter that integrates p53-dependent feedback, inflammatory signaling, and epigenetic state to shift the p53 network toward pro-survival, repair-oriented, or, in pathological contexts, pro-tumorigenic outcomes.

To summarize, the dual promoter structure of the *TP53* gene enables the generation of multiple isoforms thanks to both alternative transcription initiation and splicing mechanisms. Cellular context, cell signaling and genotoxic, mitogenic, and inflammatory stimuli, as well as genetic polymorphisms and p53 itself, finely tune p53 isoforms’ expression and p53’s tumor suppressor role. In particular, shorter isoforms generated from the P2 promoter represent important regulators of p53 activity and retain a major role in tumor progression.

### 2.2. Alternative Translation Initiation as a Modality of Regulation for p53 Isoforms

An additional method of p53 and its isoforms’ expression regulation is represented by alternative translation initiation sites. It was in fact shown that, especially the Δ40p53 isoform, but also the full-length protein, can also be produced through an Internal Ribosome Entry Site (IRES) [61,62]. Even though their mechanism of action is not completely understood, these elements are able to directly recruit ribosomes for translation initiation. The process can also be favored by the recruitment of canonical or non-canonical initiating factors, also known as IRES Trans-Acting Factors (ITAFs). Cellular proteins that have been identified as ITAFs include Polypyrimidine Tract-Binding protein (PTB) and other proteins, such as Death-Associated Protein 5 (DAP5) [63].

p53’s mRNA presents two separate IRES regions: one in the canonical 5′-UTR, encoded from exon 1 and used for the production of the full-length protein; alternatively, the other is located upstream the second AUG start site, near codon 40, and is used to produce the Δ40p53 isoform [62]. It is already established that Δ40p53 is mainly produced during the cell cycle progression from G1 to S phase [64]. Accordingly, it was seen that the IRES mediating its translation presented its maximum activity right in the G1-S transition, while the first IRES was more active during the G2-M transition [62].

IRES-driven translation is promoted by several stresses, such as nutrient deprivation, hypoxia, and DNA damage, when cap-dependent translation is compromised [65,66]. Accordingly, under stress conditions, including oncogenic-induced senescence, a switch to IRES-mediated translation of full-length p53 and Δ40p53 has been reported [67,68], which indicates a possible way for cells to increase p53’s production in order to stop cell cycle progression or induce apoptosis in response to stress. Hence, it has been demonstrated that the production of Δ40p53 from IRES is favored upon the accumulation of misfolded proteins within the Endoplasmic Reticulum via PERK-dependent Unfolded Protein Response (UPR), resulting in the induction of cell cycle arrest [69].

ITAFs also play a fundamental role in increasing IRES-mediated translation of p53 and other proteins upon stress. Cellular proteins such as PTB and DAP5, but also RPL26 (60S Ribosomal Protein L26), have been identified as positive regulators for IRES-mediated translation of p53 [70,71,72]. Upon DNA damage, RPL26 binds the 5′-UTR of p53’s mRNA, favoring its translation in order to induce cell cycle arrest [70]. A fundamental factor in IRES-mediated translation is also the availability and subcellular localization of ITAFs. For instance, upon doxorubicin treatment, PTB seems to translocate from the nucleus to the cytoplasm, increasing p53 translation in response to the stress [71]. As p53 is a fundamental tumor-suppressive protein, cancer cells may be able to decrease its action, without the appearance of mutations in the gene, through the down-regulation of some ITAFs that positively regulate its translation.

Δ40p53 is considered a negative regulator of full-length p53, as it presents impaired transcriptional activation ability, does not complex with the regulator protein MDM2 and is able to oligomerize with the full-length isoform of p53, decreasing its transcriptional activity [64]. Despite its independent tumor-suppressive action [73], in specific conditions cancer cells may be able to selectively increase this isoform through its IRES to favor survival. Indeed, high ratios of Δ40p53 in respect to the full-length protein have been shown to potentiate cell survival and aggressiveness in melanoma and breast cancer patients [40,74]. A way through which cancer cells may be able to favor Δ40p53 is through mutations or Single Nucleotide Polymorphisms (SNPs) affecting the first IRES, controlling the production of the full-length protein. In melanoma cancer patients, in fact, the presence of a SNP (C119T) in the 5′-UTR region of p53 mRNA led to a decrease in binding of the ITAF protein PTB, decreasing the activity of the first IRES and thereby the production of full-length p53 and its suppressive abilities [75]. Another way to preferentially increase the translation of Δ40p53 could be through specific ITAFs. For instance, DAP5 was found to preferentially activate the second IRES of p53 mRNA, potentially increasing the ratio between Δ40p53 and the full-length protein [72]. An over-expression of this ITAF could potentially improve cancer cells survival and aggressiveness by favoring the isoform that regulates the full-length protein.

More recently, an additional alternative translation initiation site has been found near codon 160 of p53 mRNA, emerging mainly in p53 mutant cells [76]. This element can initiate translation of Δ160p53 isoform from the mutant p53 transcript, independently from the Δ133p53 isoform, which is instead produced mainly through an internal promoter [32]. The Δ160p53 protein is one of the latest discovered isoforms of p53, and while little is known about its production and functions, evidence shows that it has a potential oncogenic role. Along with the Δ133p53 isoform, Δ160p53 also seems to work as a negative regulator of the full-length protein, as it reduces its transcriptional activator role through hetero-oligomerization [77]. According to this, cancer cells may also be able to favor these isoforms to promote tumorigenesis and cancer aggressiveness. The Δ133p53β variant was shown to drive invasiveness in both breast and colon cancer cells [78]. Furthermore, a study showed that specific gain of function mutations of p53 led to an over-expression of the shorter isoforms of the protein. Δ160p53 played a crucial role in favoring invasiveness and promoting tumorigenesis in breast cells upon mutations that disrupt the transcription of the full-length p53 mRNA [76].

Finally, a new alternative translation initiation start site has been hypothesized in codon 246. This element leads to the formation of a novel p53 isoform, called Δ246p53. This isoform seems to be induced upon DNA damage, triggering senescence, and impairing tumor formation by cancer cells. The study shows, in fact, that this isoform can down-regulate MDM2, the principal negative regulator of p53, through hetero-oligomerization with full-length protein. Additionally, they also show that Δ246p53 is able to induce p21 expression independently from full-length p53 [36]. This new discovery proposes a potential new tumor-suppressive action of p53, through this novel isoform. Understanding the specific mechanisms that trigger the production of Δ246p53 could lead to future therapies aiming at the selective induction of this isoform to potentiate the tumor-suppressive functions of p53.

Overall, these studies indicate that several isoforms of p53 are involved in tumorigenesis and cancer progression, highlighting the complex mechanisms of p53 regulation through alternative translation initiation, even in the absence of mutations. Future efforts could focus on exploring and further understanding these mechanisms to selectively target the action of the oncogenic isoforms, or the factors that favor their production, or, on the other hand, induce the tumor-suppressive isoforms.

### 2.3. Alternative Splicing as a Mechanism of Regulation for p53 Isoforms

Alternative splicing is a post-transcriptional process that enables the generation of multiple protein isoforms from a single pre-mRNA. *TP53* is subject to this mechanism, producing several isoforms that can have distinct and sometimes opposing effects on tumor suppression [31].

In *TP53*, alternative splicing can occur either at intron 2 or intron 9. Alternative splicing at intron 9 generates the full-length p53α, as well as the shorter p53β and p53γ isoforms, with the inclusion of either exon 9β or exon 9γ. Both exon 9β and exon 9γ contain stop codons that prevent the translation of exons 10 and 11 when included in the coding sequence. Conversely, alternative splicing at intron 2 results in its retention. Intron 2 contains premature stop codons, which block translation initiation from the canonical AUG1, thereby forcing translation to initiate at the downstream AUG40 codon. If no additional splicing occurs, the isoform is defined as Δ40p53α. When intron 9 is also spliced, the isoforms Δ40p53β or Δ40p53γ are produced, depending on the exon included. If splicing at intron 9 occurs concurrently with transcription initiation at the P2 promoter, the isoforms ∆133p53β, ∆133p53γ, ∆160p53β, and ∆160p53γ are generated [1,34].

Alternative splicing is regulated by RNA sequence elements and splicing factors, which control exon inclusion or exclusion in response to cellular signals. Among the key regulators of alternative splicing are the serine/arginine-rich splicing factors (SRSFs), members of the arginine-rich protein family, which bind specific cis-acting elements within pre-mRNA to promote or repress exon inclusion. By this regulation, SRSFs contribute to controlling the balance of p53 isoforms, influencing p53’s tumor-suppressive functions and its role in cancer [79].

Several SRSF family members, including SRSF1, SRSF3, and SRSF7, have been shown to directly regulate p53 isoform production.

SRSF1 is one of the first identified SR proteins and the first recognized proto-oncogene in this family. It is involved not only in splicing but also in numerous other cellular processes, such as micro-RNA (miRNA) processing, protein sumoylation, and the nucleolar-stress response [80].

In the context of *TP53*, SRSF1 promotes the exclusion of intron 9, favoring the formation of p53α. Knockdown of SRSF1 in MCF7 cells increases the levels of p53β and p53γ isoforms while reducing p53α levels, both at the mRNA and protein levels. This effect has also been observed in primary breast tumors and various other tumor cell lines, where high SRSF1 levels were associated with elevated p53α expression and undetectable levels of the p53β isoform. This highlights the importance of SRSF1, which, by influencing the balance between p53α and the shorter p53β and p53γ isoforms, modulates the p53-dependent response [31].

SRSF3, the smallest member of the twelve-protein SR family, promotes exon inclusion by binding cysteine-rich regions and recruiting additional regulatory factors. Like SRSF1, SRSF3 is considered a proto-oncogene and is over-expressed in various types of tumors.

In the context of p53, SRSF3 promotes the production of full-length p53α. Down-regulation of SRSF3 in fibroblasts undergoing replicative senescence is associated with increased p53β levels. This has been further confirmed in normal fibroblast and in various Non-Small Cell Lung Cancer (NSCLC) cell lines, where SRSF3 knockdown elevates p53β expression at both mRNA and protein levels. Mechanistically, SRSF3 inhibits p53β production by binding to a specific region within exon i9, thereby preventing its inclusion. Given the critical role of p53β in promoting cellular senescence, SRSF3-mediated repression of p53β may facilitate tumor progression by limiting pro-senescence signals. Conversely, inhibition of SRSF3 may exert tumor-suppressive effects by enhancing cellular senescence [81,82].

SRSF7 is another SR family member involved in p53 alternative splicing regulation. Unlike SRSF1 and SRSF3, which favor the production of p53α, SRSF7 promotes the formation of the p53β isoform. Notably, this effect is observed in response to ionizing radiation, suggesting a context-dependent role for SRSF7 in modulating p53 isoform expression under genotoxic stress conditions [83].

In addition to SR proteins, other splicing regulators contribute to p53 isoform control. One example is SF3B1, a component of the U2 small nuclear ribonucleoprotein (snRNP) complex. SF3B1, a core component of the U2 small nuclear ribonucleoprotein (snRNP) complex, is essential for accurate recognition of the branch point sequence during spliceosome assembly and activation [84]. Its role in the direct control of p53 splicing isoforms is less characterized compared to SR proteins, but pharmacological inhibition of SF3B1 with the specific inhibitor Pladienolide B has been shown to increase full-length p53 expression while reducing the truncated Δ133p53 variant, leading to a decreased Δ133p53/p53 ratio in cancer cells [85]. The Δ133p53 isoforms can act in a dominant-negative manner by interacting with full-length p53 and have been associated with enhanced tumor progression, even though their role heavily depends on the cellular context. Thus, the modulation of SF3B1 activity may shift the balance toward canonical p53 tumor-suppressive functions [42]. Interestingly, SF3B1 was found mutated in different types of cancer such as 15% of uveal melanoma cases, predominantly at codon R625. In uveal melanoma, mutations in SF3B1 are associated with a better prognosis, compared with tumors lacking these mutations [86]. In experimental models, co-occurrence of *SF3B1* mutation with *BAP1* loss induces cellular senescence, during which p53 is up-regulated and its downstream targets partially activated, contributing to cellular senescence [87]. Considering that p53 is usually wild-type in uveal melanoma and that *SF3B1* mutations are associated with favorable prognosis, p53 activity in these tumors may be influenced by SF3B1-mediated alternative splicing, potentially even in the absence of *BAP1* loss, although this has not been experimentally tested. Such modulation could help restrain tumor progression and maintain the less aggressive phenotype characteristic of *SF3B1*-mutant uveal melanomas.

Another regulatory mechanism influencing *TP53* splicing involves G-quadruplex (G4) structures. These secondary structures form within G-rich sequences through the assembly of four guanine bases into a G-quartet, with the stacking of multiple G-quartets generating a stable G-quadruplex. G-quadruplexes can influence splicing when located within intronic regions [88]. In *TP53*, intron 3 is enriched in guanine residues capable of forming G-quadruplexes that influence the alternative splicing of intron 2. Mutations affecting the guanines involved in G-quadruplex formation increased the presence of Δ40p53, indicating that the G4 structure normally promotes intron 2 removal. Stabilization of the G-quadruplex favors the production of full-length p53 while reducing expression of the Δ40p53 isoform, further confirming the involvement of this structure in the mechanisms regulating the alternative splicing of p53 [89].

Alternative splicing represents a crucial post-transcriptional mechanism that allows the generation of multiple isoforms with distinct biological activity. This process adds another layer of complexity to the p53 signaling pathway itself. Splicing regulators, like SRSF proteins, components of the spliceosome machinery, and RNA elements, tightly regulate p53 pre-mRNA splicing and the ratio of p53 isoforms. The p53 isoforms have distinct and sometimes opposing roles, and, consequently, understanding the mechanisms governing *TP53* alternative splicing may open new avenues for therapeutic intervention aimed at restoring a tumor-suppressive isoform balance.

### 2.4. 3′-UTRs as Post-Transcriptional Regulatory Elements of p53 Isoforms

3′-untranslated regions (3′-UTRs) are non-coding parts of mRNAs that largely contribute to post-transcriptional gene regulation. They influence mRNA stability [90,91], localization [92], and translation [93], often through sequence elements that serve as binding sites for microRNAs (miRNAs) and RNA-binding proteins (RBPs), allowing fine-tuned and context-dependent control of mRNA expression [94].

Applied to the *TP53* locus, the general principles of 3′-UTR biology suggest that, when encoded by transcripts with distinct 3′-UTRs, isoforms such as p53α, p53β, or p53γ could be regulated differently at the post-transcriptional level. Similarly, this may also apply to transcripts encoding N-terminally truncated isoforms, such as Δ133p53 or Δ40p53, which may be subjected to distinct 3′-UTR-mediated control if they harbor unique 3′-UTR regions. Given that alternative promoters and splicing generate diverse *TP53* transcripts [30], variability in 3′-UTR length or composition, which can also arise from alternative polyadenylation (APA) [95], could modulate interactions with miRNAs and RBPs. This, in turn, may influence isoform-specific expression.

However, recent genetic evidence suggests that the *TP53* locus may not fit this model as precisely as expected. By performing a deletion of the native *TP53* 3′-UTR in human and mouse cells using CRISPR/Cas9 technology, Mitschka and Mayr [96] demonstrated that the absence of the 3′-UTR has a minimal impact on the steady-state p53 mRNA or protein levels, both in basal conditions and following genotoxic stress induced by various agents. The authors proposed that, in the genomic context, 3′-UTR-mediated effects on overall p53 production were abolished by the presence of the full coding sequence, indicating a dominant influence of the latter over 3′-UTR-mediated regulation. This observation contrasts with reporter-based assays, where isolated *TP53* 3′-UTR sequences have been shown to exert measurable regulatory effects [96,97,98,99]. A possible explanation for this discrepancy may be found in the experimental context in which the 3′-UTRs are studied. Reporter assays generally isolate 3′-UTR sequences and analyze their function when placed downstream of heterologous regions encoding one or more reporter genes and are expressed under artificial regulatory elements. Therefore, despite being well-established assays, they simplify the native transcript environment. In contrast, when considered within the endogenous *TP53* locus, the 3′-UTR is part of a full-length mRNA. In this context, long-range interactions between the 5′-UTR, the coding sequence, and the 3′-UTR itself [100] may influence RNA structure and accessibility of regulatory motifs. In addition, competition for RNA-binding proteins and miRNAs, which may also bind other sites within the full-length mRNA [99], can further modulate translational efficiency. As a result, the regulatory effects attributed to the 3′-UTR and detected in reporter systems may be compensated or overridden in the context of the complete transcript.

These reporter-based studies not only present the 3′-UTR as a regulatory element for p53 expression but also identify the factors responsible for its regulation. Multiple miRNAs have been reported to bind the *TP53* 3′-UTR and reduce p53 expression, such as miR-25 and miR-30d that were shown to repress *TP53* 3′-UTR reporter activity and decrease p53 protein levels [98] or, similarly, miR-1285 [97]. These effects are highly context-dependent, consistent with miRNAs being fine-tuners of p53 expression rather than strong repressors. In an isoform-centric view, the role of miRNAs in selectively regulating specific p53 isoforms remains elusive, since the previous studies highlight the effects of miRNA interactions with the canonical *TP53* 3′-UTR [97,98].

RNA-binding proteins (RBPs) are another class of post-transcriptional regulators that have been shown to interact with the *TP53* 3′-UTR and modulate p53 expression, often in response to stress. For example, PTB (Polypyrimidine Tract-Binding protein) binds the *TP53* 3′-UTR and has been shown to regulate p53 translation and stability [99]. Some RBPs, such as HuR [101] and Hzf, can also interact and cooperate [102] with each other in modulating p53 mRNA stability and translation through interactions with its 3′-UTR, particularly under stress conditions. As for the miRNAs, the direct evidence linking specific RBPs to the regulation of individual isoforms remains limited, highlighting the need for isoform-specific studies.

miRNAs and RBPs do not act independently, and, in the context of isoform diversity, RBP-mediated regulation could contribute to differential expression of p53 isoforms if transcripts differ in 3′-UTR length or structure. For instance, the RBP–miRNA interplay at the *TP53* 3′-UTR can influence not only full-length p53 but also the Δ40p53α isoform, suggesting that specific isoforms may be differentially sensitive to RBP-mediated regulation depending on transcript context. This interplay is often competitive. Indeed, RBPs can compete with miRNAs for overlapping binding sites or remodel RNA structure to alter miRNA accessibility [99].

Apart from canonical 3′-UTR-mediated regulation, RNA secondary structures like those formed by the interaction between 3′-UTR, 5′-UTR and RBPs [100], the m6A modification which is often enriched in 3′-UTRs and is associated with cancer [103,104], and the subcellular localization, which is also regulated by the 3′-UTRs [92], of *TP53* transcripts may also influence isoform expression. Although isoform-specific evidence remains scarce, these mechanisms could potentially contribute to differential translation or stability, particularly in cancer contexts that are often characterized by dysregulated RNA modification or processing machinery.

Taken together, these observations indicate that while the *TP53* 3′-UTR may have regulatory potential, its contribution to endogenous p53 output remains uncertain and may be highly context-specific, subtle, or dependent on interactions with other transcript features and regulatory elements. Whether such regulatory effects can also influence isoform-specific expression remains an open question as well, which calls for more in-depth analysis of the individual isoforms.

## 3. p53 Isoforms’ Involvement in Cancer

p53 isoforms are clinically relevant at both the mRNA and protein level, and generally over-expressed in a variety of types of human cancers, thus making them promising cancer biomarkers. They can either promote or inhibit tumor progression through direct action or interaction with FLp53α [26].

### 3.1. Full-Length p53 Isoforms

Full-length p53 isoforms are generated from the P1 promoter and can be spliced at C-terminus originating the α, -β, -γ isoforms. FLp53α is the canonical version of the protein, and it is responsible for the known and well-established p53 tumor suppressor functions described in the Section 1. p53β and p53γ isoforms mostly demonstrate tumor suppressor functions among multiple cancer types and are suggested to influence treatment response and therapy outcome. Their expression levels are regulated by the proteasome and appear to be, at least partially, MDM2-independent [105]. They lack the C-terminus MDM2-binding domain, making them less susceptible to MDM2-mediated degradation. Indeed, p53β and p53γ isoforms exhibit high susceptibility to nonsense-mediated decay (NMD), a process that regulates aberrant mRNA stability. For these reasons, tumors that present high MDM2 expression together with *TP53* mutations, NMD inhibition favors p53β and p53γ isoforms up-regulation and restore activation of p53 pathways. In this scenario, expression of these isoforms can rescue p53 pathway activation and tumor suppressor activities [106]. p53β differs from FLp53 because it lacks the carboxy-terminal oligomerization domain due to alternative mRNA splicing. It promotes apoptosis and senescence in a p53α-dependent and p53α-independent manner, even if to a lesser extent than p53α. In fact, p53β binds prominently to the pro-apoptotic *BAX* promoter, while it binds with poor affinity to the *MDM2* promoter. Moreover, in conditions where cellular stresses are absent, p53β can enhance p53 transcriptional activity on the *CDKN1A/p21* and *BAX* promoters [44]. p53β can also form a complex and hetero-tetramerize with FLp53, hence regulating its transcriptional and tumor suppressor activity, influencing the selection of the target gene that is regulated and transcribed. On the contrary, p53γ enhances p53 transcriptional activity on the *BAX* promoter but not the *CDKN1A/p21* promoter. p53γ can also regulate transcription in a p53-independent manner [107,108]. Furthermore, p53β appears to be highly expressed in cells during replicative senescence. It inhibits cell proliferation and induces cellular senescence cooperating with FLp53, as observed in colon adenomas with senescent phenotypes. Studies demonstrated that altered expression of this isoform contributes to senescent phenotype in premalignant lesions and to senescent barrier escape, as well as malignant progression [107]. p53β is over-expressed in renal cell carcinomas, where it represents a good predictor of cancer progression, and melanomas, and its mRNA levels are associated with tumor stages in these cancer types [109,110,111]. In addition, it correlates also with ovarian cancer prognosis and, in patients with functionally active p53, expression of p53β isoform correlates with worse recurrence-free survival and was associated with adverse clinic-pathological markers [112]. Expression levels of p53β and p53γ were found to correlate with increased response to chemotherapy treatment such as camptothecin and doxorubicin in H1299 and SAOS-2 cells. Chemotherapy treatment led to degradation of p53γ isoform, mostly localized in the cytoplasm, while nuclear localized p53β showed enhanced stability upon treatment. Lastly, in vivo findings highlight that p53β and p53γ cause an earlier tumor growth initiation, promoting metabolic adaptation and tumor growth advantage through p21 modulation [105]. Expression of both p53β and p53γ is lost in 60% of human breast cancers. Recently, it was shown that elevated cytoplasmic levels of p53β were associated with poorer prognosis in breast cancer [113]. Despite this, differential expression of these two isoforms may also explain the inconsistent relationship between p53 mutation and cancer progression and prognosis across patients. To note, in breast cancers harboring p53 mutations, the expression of p53γ abrogates the poor prognostic effect associated with p53 mutations, with decreased risk of recurrence and survival rate comparable to patients presenting wild-type p53. On the contrary, breast cancer patients expressing mutated p53 not accompanied by p53γ expression, present poor prognosis. In the same patients, p53β expression was associated with ER expression in primary breast cancers, but not with disease outcome [114]. In p53 mutated gastric cancers, p53β expression favors the inhibition of cancer cell proliferation [115]. Instead, elevated levels of p53γ isoform are associated with decreased progression-free-survival in uterine serous carcinoma [116]. The over-expression of p53β and p53γ is also correlated with increased responses to chemotherapy in acute myeloid leukemia [117]. In head and neck squamous cell carcinoma (HNSCC), p53β and p53γ were the most detectable p53 isoforms along the canonical p53α in both tumor and normal adjacent tissues [118]. Lastly, in a cohort of multiple myeloma patients, high expression of p53β/γ was correlated with worst prognosis and survival. On the contrary, patients with high levels of p53α and low levels of p53β/γ expression exhibited improved sensitivity to valproic acid treatment [119]. Noteworthily, very recently it has been shown that p53γ isoform, on top of Δ133p53 and Δ160p53 (see below), has an elevated propensity to form aggregates thanks to a novel aggregation-prone region in the p53γ-specific region which is shared with the p53 isoforms starting from the downstream ATGs [120].

Interestingly, p53 isoforms can be “mutated” or altered, thus contributing to further alteration of p53 signaling pathways and susceptibility to cancer development. It has been observed that heterozygous stop-lost variant of p53β isoforms (p.*342Serext*17) leads to 17-aa (SREHENGSMTLPDTDAT) extension of this isoform, increasing the oligomerization with FLp53 and dysregulating p53’s transcriptional target expression. Its expression correlates with autosomal dominant cancer syndrome and contributes to the increased susceptibility to colorectal, breast, and papillary thyroid cancers [121]. This is the first study reporting a mutation in a p53 isoform-specific coding exon that can be associated with a cancer predisposition.

All together, these pieces of evidence highlight the fundamental role of full length p53 isoforms expression in cancer onset and progression. Even though C-terminally truncated p53β and p53γ isoforms show altered and more often tumor-suppressive behavior, their role seems to be context- and cell/tissue-dependent. Further studies will be needed to clarify the impact of these isoforms in FLp53 activity regulation and response to therapy.

### 3.2. Δ40p53 Isoforms

Δ40p53 isoforms can be generated by alternative initiation of translation at codon 40 of the *TP53* gene or by alternative splicing that retains intron 2, which contains a stop codon followed by an internal ribosome entry site (IRES), allowing translation to restart at codon 40. In contrast to full-length p53, Δ40p53 isoforms lack the first TAD1, including the MDM2-binding site, but retain the second TAD2, leading to an extended protein half-life and the ability to transactivate a different set of target genes [122]. In particular, Δ40p53 can induce apoptotic genes that are inaccessible to FLp53 [66,123] and can form hetero-tetramers with FLp53 with a higher propensity compared to FLp53 alone [124]. Δ40p53 function is dose-dependent: at low levels, Δ40p53 can stimulate FLp53-dependent transactivation [125], while at higher levels, Δ40p53 can exert a dominant-negative effect on FLp53, repressing the transactivation of FLp53-target genes, such as p21 or MDM2 [64]. Moreover, Δ40p53 can independently regulate various coding and non-coding targets, including lncRNAs and miRNAs, which affect cell cycle, adaptation to stress, and stemness mechanisms [122]. In the context of stem cell biology, in pluripotent and progenitor cells, Δ40p53 functions as a central regulator of the balance between self-renewal and differentiation. Haploinsufficiency for Δ40p53 triggers loss of pluripotency markers (NANOG, SSEA-1), down-regulation of proliferation, and acquisition of somatic cell cycle characteristics, while its over-expression prolongs self-renewal and blocks differentiation by modulating FLp53 activity at key pluripotency targets, particularly NANOG and the IGF-1 receptor (IGF-1R) [126]. Moreover, Δ40p53α is involved in the regulation of senescence and aging. Its ectopic expression resulted in a progeroid phenotype in mice, including accelerated aging, reduced bone density, and loss of fertility. This is associated with the up-regulation of IGF-1 signaling, cellular senescence, and altered glucose homeostasis [126].

In the context of human cancer, the functions attributed to the Δ40p53 isoforms show considerable variability according to the cancer type, the *TP53* mutational status, and the relative ratios of the isoforms, and they are closely related to cancer stem cell (CSC) biology and treatment responses. In breast cancer, the expression of Δ40p53 is significantly up-regulated in the tumor tissue compared to normal breast tissue, with high expression associated with aggressive breast cancer subtypes, particularly triple-negative breast cancer [127]. Moreover, a high Δ40p53:FLp53 ratio correlates with poor disease-free survival and promotes CSC phenotypes [128]. Specifically, Δ40p53 colocalizes with the pluripotency transcription factors SOX2, OCT4, and NANOG in breast cancer cell lines, promoting increased expression of these stemness markers, enhanced mammosphere and colony-forming ability, decreased expression of the anti-stemness miRNAs miR-145 and miR-200, increased tumor growth, microvascular area, and resistance to doxorubicin in vivo [74]. Thus, these data indicate that Δ40p53 is a critical mediator of CSC maintenance and chemoresistance in breast cancer, suggesting that therapeutic strategies combining Δ40p53-specific inhibition with conventional chemotherapy may effectively target cancer stemness. In glioblastoma, Δ40p53α is detected in tumor tissue, where it promotes cell proliferation, while it is absent in normal cerebral cortex [129]. In renal cell carcinoma, expression of Δ40p53α is elevated in mutant *TP53* tumors compared to wild-type *TP53* tumors [130]. In melanoma, Δ40p53β expression is reduced in tumor tissue, but in vemurafenib-resistant melanoma cells, there is an increased expression of Δ40p53β and a decreased expression of FLp53β [131], suggesting that shifts in isoforms expression are implicated in the acquisition of therapeutic resistance. Furthermore, recent evidence demonstrates that Δ40p53 directly binds to an internal promoter of netrin-1 gene via a canonical p53-binding site, thereby up-regulating both netrin-1 and its dependence receptor UNC5B independently of full-length p53. This activation renders lung, colon, and melanoma cancer cells addicted to netrin-1 for survival. Notably, Δ40p53 expression positively correlates with netrin-1 levels in melanoma and colorectal cancer biopsies, highlighting its context-dependent pro-oncogenic role and potential as a biomarker for netrin-1-targeted therapies [132]. Very recently, Pal and colleagues performed a mIRnome analysis in cells over-expressing Δ40p53α or FLp53α and identified miR-4671-5p as Δ40p53α target. Specifically, by down-regulating miR-4671-5p, Δ40p53α is able to favor S-phase progression [133]. In contrast to these potentially pro-tumorigenic functions, Δ40p53 isoforms can exert opposing roles in other cancers. In particular, in ovarian cancer, Δ40p53α expression relates to a favorable clinical outcome: patients with wild-type *TP53* and high levels of Δ40p53α exhibit increased disease-free survival [134], and a similar correlation is observed in *TP53* wild-type serous ovarian cancer, though without impact on overall survival [112].

Altogether, these findings highlight that Δ40p53 isoforms are promising biomarkers and therapeutic targets in oncology, and that their role in cancer is dependent on the cellular context, the tissue microenvironment, *TP53* mutational status, and ratios with FLp53.

### 3.3. Δ133p53 Isoforms

Unlike static *TP53* mutations, Δ133p53 levels reflect the tumor’s dynamic adaptive response.

While the presence of WT p53 in cancer tissues typically signals a favorable prognosis due to its tumor-suppressive ability, the concomitant over-expression of the Δ133p53 isoform acts as a dominant-negative inhibitor, reducing this response. In glioblastoma and serous ovarian carcinoma, a high Δ133p53/FLp53 ratio has been consistently correlated with shorter progression-free survival and overall survival [135]. This prognostic factor originates from the Δ133p53 isoform’s ability to promote a pro-survival state even in the presence of genotoxic chemotherapy [55].

Recent clinical data have highlighted the prognostic significance of the Δ133p53 isoform also in ovarian cancer, mainly during advanced-stage disease. Indeed, in a prospective multicenter study of 154 patients diagnosed with stage III-IV serous ovarian cancer, favorable outcomes were observed in p53 mutant cases. Specifically, high expression of Δ133p53 correlated with significantly prolonged recurrence-free survival (HR = 0.571; *p* = 0.016) and overall survival (HR = 0.365, *p* = 0.004). These results were validated by parallel analyses in advanced serous ovarian carcinoma (HGSOC), in which Δ133p53 mRNA levels served as an independent biomarker for improved overall survival (HR = 0.422; *p* = 0.018). Collectively, these data suggest that Δ133p53 expression confers a survival advantage and has broad clinical utility across ovarian histological subtypes, acting as an important biomarker for risk stratification [135].

In prostate cancer, Δ133p53β expression was specifically used as a biomarker for elevated immune-cell infiltration. Indeed, it had been shown that Δ133p53 could contribute to oncogenesis by promoting inflammation. In vivo models, particularly those expressing the functional mimic of human Δ133p53, Δ122p53, exhibited a systemic inflammatory phenotype characterized by elevated serum cytokines; this pro-inflammatory state appears to be driven by the canonical NF-κb pathway. This increase has been observed in both peripheral blood mononuclear cells from Δ122p53 mice and human carcinoma cells transfected with Δ133p53α. These results indicate a link between p53 isoform dysregulation and broader immune modulation in the tumor microenvironment [136].

Δ133p53β appears to be increased in aggressive primary tumors. This data confirms the propensity of certain cancers, specifically lung, breast, and prostate, to be able to migrate and form metastasis into the brain [137]. In prostate cancer, seeing this isoform alone gives you an 88% accuracy rate in predicting aggressive disease. In breast cancer, this effect is even more ambiguous; Δ133p53β was found elevated in patients with good prognosis, yet it ended up being the most significant predictor of death and recurrence. Essentially, the presence of Δ133p53β was directly linked to a shorter time for primary tumors to spread to the brain. This is since it causes cells to lose adhesive structures and adopt a rounded amoeboid morphology, which is optimized for squeezing through tissues. Both human Δ133p53β and its mouse equivalent (Δ122p53β) significantly boost cell migration, even in cells that are typically non-invasive. In laboratory tests (trans-well assays) mimicking the restrictive blood–brain barrier (endothelial cells and astrocytes), cells with elevated levels of this isoform showed a superior ability to migrate through [78]. It achieves this by enhancing the production of several notorious “bad actor” proteins. By increasing the expression of cell-surface receptors like EGFR, c-MET, and VEGFR, Δ133p53β essentially equips the cell with the tools it needs to survive the journey and thrive once it reaches the brain [78].

Moreover, pre-clinical and clinical studies evaluated ∆133p53 mRNA expression, signaling pathways, and histopathology in a cohort of 35 colorectal cancer patients. Interestingly, tumors that over-expressed ∆133p53 showed enrichment of GPCR signaling upstream of the JAK/STAT and RhoA/ROCK pathways, mirroring the ∆122p53 mouse models, and were characterized by reduced lymphocyte infiltration and shorter disease-free survival. These observations were associated with low immune cell infiltration and poorer prognosis in colorectal cancer [138]. The ∆133p53 modulates cytokine profiles in a tissue-specific manner, thereby altering immune cell trafficking within the tumor microenvironment. Furthermore, in colorectal cancers, ∆133p53 forms appeared not to be involved in benign tumor formation, while they correlated with progression from benign to malignant tumors. Indeed, ∆133p53 was found to be up-regulated at the mRNA level during adenoma-to-carcinoma progression, whereas it was down-regulated in colon adenomas compared with non-tumor cells [42].

As mentioned above, a comparable up-regulation of ∆133p53 total mRNAs has been observed in gastric tumors, especially those driven by pathogens such as *Helicobacter pylori* [57]. Here, ∆133p53 isoforms have been shown to promote cancer cell survival and to correlate with the expression of the NF-κB p65 subunit, further increasing malignant gastric cell growth. *H. pylori* itself up-regulates the expression of ∆133p53 isoforms mRNAs and, in turn, ∆133p53 inhibits FLp53 activity, inducing the NF-κB pathway to decrease apoptosis [139]. In addition, increased ∆133p53 and decreased p53β expression seem to represent a trend during gastric carcinogenesis from superficial gastritis to atrophic gastritis and, lastly, gastric adenocarcinoma [140].

Elevated levels of Δ133p53β were linked with glioblastomas particularly enriched in macrophage content, while the normal brain expressed low levels of Δ133p53. Specifically, Δ133p53β was particularly high in telomerase-positive tumors expressing wild-type p53. Researchers used RNAscope and ImmunoHistoChemistry (IHC, using the KJC8 antibody) to detect Δ133p53β in 20 glioblastoma samples, with the aim of identifying cells that express the isoform. One of the most significant findings was the presence of Δ133p53β in pseudo-palisading cells, particularly in the tumor. These cells are essentially the “front lines” of the tumor, often consisting of malignant cells migrating away from necrotic zones. Overall, despite the limited sample numerosity, the authors identified Δ133p53β as a p53 isoform associated with immunosuppression and chemoresistance in glioblastoma [141].

In tumors where *TP53* mutations were rare, recent evidence indicated that the expression of truncated p53 isoforms, particularly the Δ133p53 variants, was a primary driver of p53 pathway dysfunction. As we discussed before, the Δ133p53 variant can physically associate with FLp53α to form hetero-oligomers. This interaction rapidly triggers protein aggregation, which traps functional p53 and stops it from binding to promoters of key target genes like p21, BAX, and PUMA. But it is more than just blocking the FLp53α isoforms that actively foster a pro-survival environment. For example, Δ133p53β has been shown to promote members of the BCL-2 family, thereby shielding cancer cells from DNA damage-induced death. Indeed, Δ133p53 isoforms are frequently expressed in both uveal and cutaneous melanoma despite the predominance of wild-type *TP53*. Studies show that the short Δ133/Δ160p53 variants promote cell proliferation and blunt cell death responses to standard therapies such as cisplatin or proton beam irradiation. Comparative analysis shows a significant up-regulation of the Δ133p53 variant in metastatic UM cell lines relative to their primary tumor counterparts. In a clinical cohort, elevated levels of Δ133p53γ correlate with increased tumor basal dimensions and more aggressive histological subtypes, suggesting its utility as a biomarker for high-risk patients [43]. Moreover, high Δ133p53β expression levels were associated with a poorer prognosis in a cohort of metastatic cutaneous melanoma patients [131]. Furthermore, Δ133p53 isoforms have been linked to acquired resistance to treatments targeting MAPK; their up-regulation in resistant melanoma cells suggests they play a functional role in evading BRAF/MEK inhibition [142]. All of these factors work together to modify p53 activity, tipping the scales toward tumor growth, treatment resistance, and unfavorable clinical outcomes.

This pro-survival promotion, while seemingly at odds with senescence as a barrier to carcinogenesis, highlights a context-dependent duality: oncogenic in neoplastic settings, yet protective against genotoxic stress in healthy tissue. Δ133p53α coordinates DNA stability via both p53-dependent and independent pathways. It serves as a balance between canonical p53α—which typically suppresses double-strand break (DSB) repair in favor of apoptosis—by up-regulating vital repair factors such as RAD51 and RAD52 (via Homologous Recombination—HR), and LIG4 (via Non-Homologous End Joining—NHEJ) through its interaction with p73. Additionally, Δ133p53α can neutralize the pro-senescent action of p53α, directing the cell to survival and repair [143]. Moreover, given that it has been established that FLp53α can interact with DNA polymerase γ in the mitochondria to stimulate BER repair pathway, Δ133p53 was demonstrated to counteract this function by directly interacting with FLp53α, leading to impaired mitochondrial BER [144]. Additionally, Δ133p53α was shown to negatively impact the homology-directed DNA damage tolerance pathway, where FLp53α interacts with TLS DNA polymerase iota (DNA polι) to bypass replication barriers, a characteristic that is also common to p53β, Δ40p53, and Δ160p53α, and, in this way, decreases FLp53α tumor-suppressive functions, favoring genomic instability [145].

One of the most compelling aspects of Δ133p53α is its growing significance in neuroprotection. This isoform reduces oxidative stress and DNA damage by blocking 5-Lipoxygenase (5-LOX), a crucial enzyme linked to the onset of Alzheimer’s disease. This observation implies that Δ133p53α-mediated DNA repair is essential for both internal defense against chronic neurodegeneration (linked to tau hyperphosphorylation and amyloid formation) and recovery from external genotoxic stress. Consequently, Δ133p53α represents a promising therapeutic target for improving cellular resilience in the aging brain [42].

In summary, the body of data points to Δ133p53 as a dynamic and powerful regulator of the cellular adaptive response rather than as a passive consequence of the *TP53* gene. In cancer, it contributes to the development of aggressive malignancies, while in healthy and aged tissues, it plays a vital role in maintaining cellular integrity. 

Clinically, these observations translate Δ133p53 into a potent independent biomarker that, frequently overruling positive prognostic indications, predicts brain metastases and rapid recurrence with high statistical accuracy in the oncogenic context. The isoform-specific regulation of the p53 pathway may be the future therapeutic challenge. While elevating Δ133p53α levels appears to support DNA repair and longevity in the context of chronic neurodegeneration, researchers must seek effective therapeutic options to selectively block the pro-invasive Δ133p53β variants in primary indolent or low-grade tumors to impede metastatic progression. The next generation of precision medicine must comprehend this fine balance between cancer and survival.

### 3.4. Δ160p53 Isoforms

The Δ160p53 isoforms represent the shortest variants of the p53 protein family. They are generated through alternative translational initiation from an internal ATG codon located at position 160 within the Δ133p53 transcript, which originates from the alternative promoter P2 located in intron 4 of the *TP53* gene [32]. Following initiation at ATG160, Δ160p53 isoforms lack the first N-terminal 159 residues corresponding to both the TAD1 and TAD2, the PRD and the entire first conserved cysteine box of the DNA-binding domain [30,146]. Consequently, Δ160p53 isoforms lack the structural and functional elements required for classical p53-mediated transcriptional activation on canonical p53 response elements.

As for other p53 isoforms, alternative splicing of intron 9 of *TP53* gene generates three distinct C-terminal Δ160p53 variants. Complete excision of intron 9 gives rise to Δ160p53α, which retains the canonical C-terminal region, including the OD and the negative regulatory α domain. In contrast, alternative inclusion of exon-9β or -9γ generates Δ160p53β or Δ160p53γ, respectively, in which the OD is truncated in short, isoform-specific C-terminal peptides of 10 or 15 amino acids, respectively [30,147].

Despite their lack of transactivation domains, Δ160p53 isoforms preserve at least part of the oligomerization domain, enabling direct physical interaction with other p53 family members. In particular, Δ160p53α has been shown to exert a dominant-negative effect on wild-type FLp53 through hetero-oligomerization and co-aggregation. When it is present in excess to FLp53 within tetramers, Δ160p53α sequesters transcriptionally competent FLp53 into inactive aggregates, resulting in FLp53’s impaired DNA binding and reduced transcriptional activity [148]. Similar observations were also determined by Tomas and collaborators, demonstrating that, on top of the aggregation propensity, Δ160p53α (unlike FLp53α) adopted a mutant-like conformation itself and the resulting hetero-tetramer between Δ160p53α and FLp53α was dysfunctional, despite the increased stability of FLp53α [77]. This aggregation-mediated inactivation mechanism provides a functional explanation for the paradoxical p53 pathway suppression in tumors retaining wild-type *TP53* and identifies Δ160p53 as a non-genetic modulator of p53 tumor suppressor function.

Compared with FLp53 and other N-terminally truncated p53 isoforms, the clinical characterization of Δ160p53 remains limited. Nevertheless, accumulating experimental evidence supports a biological and clinically relevant role for Δ160p53—predominantly the Δ160p53α isoform—in cancer aggressiveness, therapy resistance and functional suppression of the p53 pathway.

The first experimental evidence linking Δ160p53 to a cancer-promoting role was provided by Candeias and colleagues. Using genetically controlled p53-null and mutant p53 models, the authors demonstrated that Δ160p53α expression confers p53 mutant-like gain-of-function phenotypes, including enhanced cell survival, sustained proliferative ability, and resistance to genotoxic stress through attenuation of apoptotic responses. Importantly, Δ160p53α was shown to be required for the oncogenic potential of mutant p53, such as increased cellular adhesion, invasive behavior, and disruption of three-dimensional tissue architecture, as suppression of Δ160p53 translation effectively abolished these pro-tumorigenic traits [76]. These findings establish Δ160p53α as an oncogenic modulator of tumor behavior downstream of both mutant and truncated p53 signaling, despite lacking canonical transactivation potential.

Expanding these observations to tumor contexts that largely retain wild-type *TP53*, Tadijan and co-workers characterized the role of Δ160p53 isoform in the context of cutaneous melanoma, identifying them as functional determinants of cancer aggressiveness. Among the analyzed p53 isoforms, Δ160p53α emerged as the most variably expressed across melanoma-derived cell lines. Functionally, while the stable over-expression of all Δ160p53 variants (α, β, γ) stimulate cellular proliferation in a p53-null background, the α and β isoforms specifically drive cell migration and are recruited to the chromatin compartment, suggesting a role in direct transcriptional modulation that can be linked to their pro-tumorigenic role [110]. Overall, these data implicate Δ160p53 isoforms as functional drivers of melanoma aggressiveness independently of the *TP53* mutational status. These observations suggest that elevated Δ160p53 isoforms expression may identify tumors with increased invasive potential in a disease currently lacking robust prognostic biomarkers.

The clinical relevance of Δ160p53 is further supported by the recent work on uveal melanoma, a malignancy that almost invariably retains wild-type *TP53* (mutation rate below 5%). In this context, Δ160p53α was identified as one of the predominant truncated p53 isoforms variably expressed across multiple uveal melanoma cell lines. Interestingly, the functional silencing of Δ160p53 appeared to enhance the sensitivity to DNA-damaging treatments in vitro, including cisplatin and proton-beam irradiation, directly linking Δ160p53 expression to therapy resistance, a central issue in clinical oncology. Moreover, the analysis of patient-derived samples revealed higher Δ133/Δ160p53 expression (they share the same transcripts as mentioned above) in tumors characterized by high-risk histopathological features and metastatic potential. Although limited cohort size precluded definitive statistical conclusions, these observations suggest a possible association between Δ160p53 expression and poor clinical outcome, reinforcing its potential utility as a biomarker of tumor aggressiveness [43].

Notably, the clinical impact of Δ160p53 expression appears to be context dependent. In multiple myeloma, high expression of short p53 isoforms, including Δ160p53, has been associated with improved overall survival in patients with high-risk cytogenetic profiles [119]. These findings suggest that, in specific cellular and genetic contexts, elevated levels of truncated p53 isoforms may reflect adaptive or compensatory responses rather than purely oncogenic functions, underscoring the complexity of p53 isoform biology in human cancer.

Collectively, available evidence positions Δ160p53α as an emerging cancer biomarker of tumor aggressiveness, therapy resistance, and functional impairment of p53 signaling. While large-scale clinical validation is still lacking, these findings support the inclusion of Δ160p53α among p53 isoforms of diagnostic, prognostic, and therapeutic relevance. Rather than serving as a classical diagnostic marker, Δ160p53 expression may identify a subset of tumors in which p53 signaling is compromised through non-genetic mechanisms despite the presence of wild-type *TP53*, thereby refining prognostic stratification beyond *TP53* mutational status alone. This concept is in line with broader analyses of p53 isoforms as cancer biomarkers and therapeutic targets [26], highlighting the need to move beyond *TP53* mutational status alone.

In contrast, the biological and clinical significance of Δ160p53β and Δ160p53γ remains largely unexplored, representing an important area for future investigations. 

Lastly, the group led by Prof. Jean-Christophe Bourdon (University of Dundee, Scotland, UK) has developed monoclonal antibodies (i.e., KJC76, and KJC77) able to specifically recognize Δ160p53 p53 isoforms which work well in Western blotting and other applications, making the detection of these isoforms easier and more accessible by researchers not specifically involved in the field.

**Figure 2:**
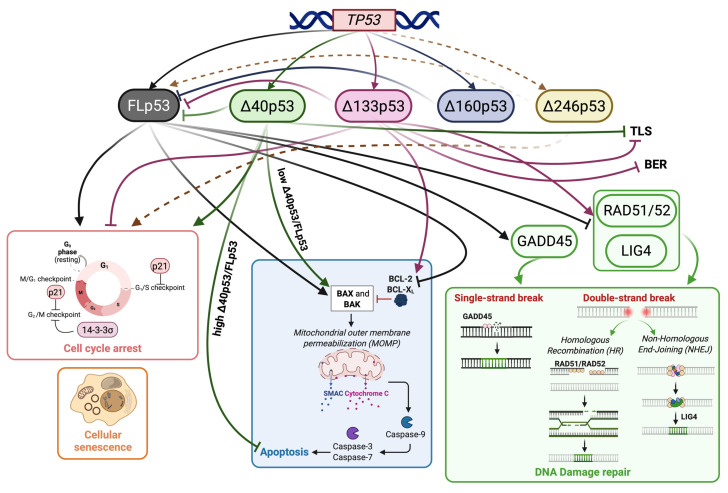
The diverse functions of p53 isoforms in cancer cells. Each isoform retains different functions. Full-length p53 (FLp53) is able to induce cell cycle arrest and single-strand break damage repair (p21, 14-3-3σ, GADD45); it also drives apoptosis, activating pro-apoptotic proteins (BAX and PIDD, p53-Induced protein with Death Domain) while inhibiting anti-apoptotic proteins (BCL-2, BCL-xL). Δ40p53 independently activates 14-3-3σ but modulates FLp53: at a high ratio it is able to inhibit apoptosis, whereas at a low ratio, it induces pro-apoptotic proteins. Δ133p53 is a negative regulator of the full-length protein, and it induces double-strand break repair through the activation of RAD51, RAD52, and LIG4. It can inhibit the activation of p21 while favoring the production of anti-apoptotic proteins. Δ133p53 also negatively impacts mitochondrial BER and TLS. Not much is known about the functions of the Δ160p53 isoform, and most studies link its oncogenic functions to its antagonistic action to the full-length protein. The latest hypothesized isoform, Δ246p53, instead, seems to be an active modulator of FLp53, which, along with its possible induction of p21, indicates a possible tumor-suppressive role. Created with Biorender.com.

The mentioned role of p53 isoforms in cancer are schematized in Figure 2, and their impact on specific cancer types are summarized in Table 1.

## 4. Effective Tools to Determine the Expression and Biological Activities of p53 Isoforms

The studies regarding detection and quantification of p53 isoforms at both mRNA and protein levels rely on specific tools (primers and antibodies) and techniques developed to overcome the challenging measurement of p53 isoforms activities. Because of conserved protein regions among the different isoforms, along with multiple layers of transcriptional and post-transcriptional regulation in their generation, specific antibodies and primers are needed to specifically detect the known p53 isoforms.

**Figure 3:**
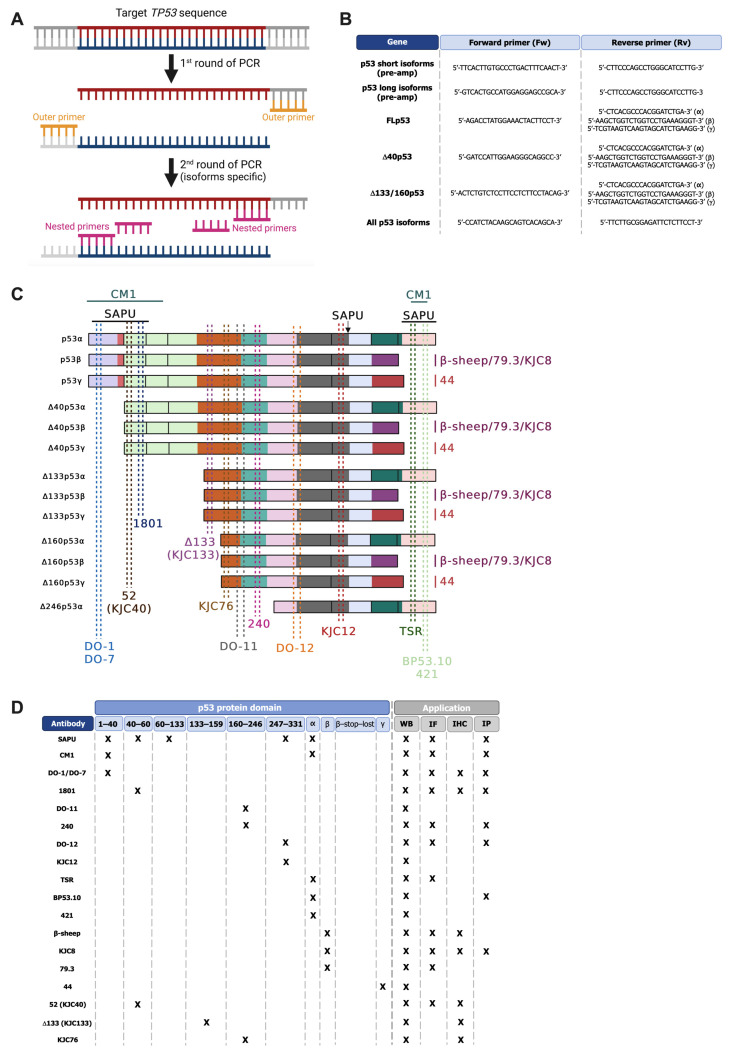
Detection methods for p53 isoforms. (**A**) A schematic representation of nested RT-qPCR approach for the quantification of p53 isoforms’ mRNA. (**B**) List of primers used for p53 isoforms’ mRNA amplification. (**C**) An illustration of antibodies developed for p53 isoforms protein detection. Dotted lines correspond to the protein region recognized by each antibody. (**D**) List of p53 isoforms specific antibodies, with the relative binding region and the technical application. WB = Western Blot; IF = Immunofluorescence; IHC = Immunohistochemistry; IP = Immunoprecipitation.

RT-qPCR represents the most reliable method for the quantification and detection of p53 isoforms. The problem of the measurement of each single isoform mRNA lies in the fact that p53 isoforms differ contemporarily at the 5′- and 3′-end. Originally, RT-qPCR approaches were based on the detection of only groups of isoforms: FLp53 or Δ133/160p53 and alpha, beta or gamma isoforms [152]. Among the different protocols developed for detecting p53 isoforms, nested PCR is the most valuable. It is based on two sequential amplification reactions, each of which is carried out with a different set of primers. The first amplification reaction generates the template for the second PCR, while the second uses internal nested primers that ensure the amplification of a specific region (Figure 3A) [30,131]. In the case of the *TP53* gene, two distinct sets of outer primers have been created. Hence, through the first reaction, it is possible to amplify a region common to all the short isoforms (Δ133/160p53) or to all the long p53 (FLp53 and Δ40p53) isoforms. Then, several sets of primers have been designed to quantify specific p53 isoforms’ mRNAs (Figure 3B). Additionally, an assay with long amplicons using droplet digital PCR (ddPCR) has been recently designed to detect some of the p53 isoforms in a robust and reliable manner [153].

However, the quantification of p53 isoforms’ mRNAs retains some limitations. Indeed, there is no specific set of primers that can distinguish between Δ133p53 and Δ160p53 isoforms, as their generation is due to post-transcriptional regulation. For the same reason, there is frequently no direct correlation between p53 isoform mRNA levels and p53 protein isoforms, so mRNA quantification alone is not sufficient to characterize p53 isoforms’ expression in cell lines and tumor samples.

For this reason, several mouse, sheep, and rabbit monoclonal antibodies have been developed to measure endogenous p53 protein isoforms (Figure 3C) through western blotting (WB), immunofluorescence (IF), and immunohistochemistry (IHC) techniques [30,31,44,154]. Each antibody recognizes a specific epitope that defines its specificity and has been determined by an ELISA approach performed on an epitope-mapping peptide library. Some antibodies, such as SAPU, KJC12, and DO-12 can detect all p53 isoforms, while others are specific for the α or β forms (Figure 3D). Interestingly, groups of isoform-specific antibodies have also been designed: 52 recognizes only Δ40p53 variants, while Δ133 and KJC76 are able to specifically detect Δ133p53 and Δ160p53 isoforms, respectively. Unfortunately, some antibodies work well for several applications, whereas others are suitable only for a limited number of experimental techniques. Moreover, the main limitation is that post-translational modifications within recognized epitopes can prevent p53 antibodies from binding to the target site. Hence, the best approach could be represented by the use of different antibodies that detect and characterize p53 isoforms in cells and tissues [30]. Further research is needed to develop more advanced and specific methods for detecting p53 isoforms to disentangle the p53 isoforms’ code in cancer.

## 5. Therapeutic Strategies Targeting p53 Isoforms

p53 isoforms offer targeted therapeutic opportunities due to their isoform-specific regulation and differential expression in cancer. Yet, isoform-selective strategies remain scarce. Recent studies have suggested potential future therapies based on the identified interaction partners or on specific novel functions of certain p53 isoforms. These therapeutic approaches include netrin-1 blockade through monoclonal antibodies (NP137) for Δ40p53-driven tumors [132], siRNA silencing of Δ160p53 to restore treatment sensitivity in uveal melanoma [43], and aggregation inhibitors targeting Δ133p53α and Δ160p53α’s stability [148].

Since some isoforms are produced via alternative translation initiation sites (Δ40p53, Δ160p53, and Δ246p53), future therapies could also focus on regulating this, acting specifically on the IRES elements of the p53 mRNA. For instance, the protein DAP5, an ITAF preferentially activating the Δ40p53’s IRES, could potentially be used as a target in Δ40p53-driven tumors [72]. However, further studies are needed to assess the potential effectiveness of siRNAs or to identify possible small-molecule inhibitors.

Promising studies also focus on aberrant alternative splicing, which is considered a hallmark of cancer [155]. Since, as previously discussed, p53 also undergoes this process in cancer cells, different therapeutic approaches may be employed to stop the formation of oncogenic isoforms. In this context, small-molecule inhibitors have shown the most promising results. The majority of these inhibitors targets the SF3B subcomplex of the U2 snRNP to modulate alternative splicing and lead to cancer cell growth arrest [156]. For instance, the inhibitor of the SF3B1 subunit Pladienolide B has been found to increase apoptosis through preferential production of pro-apoptotic splice variants, such as full-length p53, over the oncogenic isoforms, such as Δ133p53 [85].

Additionally, small molecules that affect SRSFs may be able to redirect isoform production for therapeutic benefit [157]. Specific inhibitors of the splicing factor SRSF3, such as SFI003, have been shown to lead to an alteration of the splicing pattern of p53 mRNA. These inhibitors are able to decrease p53α expression, while increasing p53β levels, leading to tumor suppression and cellular senescence, thus representing possible future therapies [158].

An alternative approach to target aberrant alternative splicing is the use of splice-switching antisense oligonucleotides (SSOs). These short nucleotide chains are chemically modified RNA molecules that bind the target pre-mRNA at a specific site, interfering with or redirecting its splicing [156]. While the use of these molecules on the p53 pre-mRNA has yet to be explored, evidence from other cases, such as Bcl-x pre-mRNA [159], suggests a possible future use of SSOs on other aberrant splicing events, highlighting their potential in cancer therapy. Alternatively, antisense oligonucleotides (ASOs) could also be employed in a context-dependent manner to target the oncogenic shorter isoforms of p53 (Δ133p53, Δ160p53). However, in this case, the ASO design could be technically challenging, as it would need to match the sequence transcribed from the P2 region in order to avoid off-target effects on the longer isoforms of the protein.

Collectively, p53 isoform dysregulation offers multiple therapeutic intercepts across translation, splicing, and isoform-specific silencing, warranting isoform profiling as a possible treatment selection strategy in precision oncology.

## 6. Conclusions

To summarize, p53 isoforms are undoubtedly critical players in cancer aggressiveness and progression. Specific subtypes of p53 isoforms can enhance or support FLp53 tumor suppressor activities, but, in parallel, others can promote angiogenesis, immune evasion, migration, invasion, and metastasis alone or by counteracting the functions of FLp53 when co-expressed. Therefore, in clinical settings the expression analysis of p53 isoforms may reveal critical information about prognosis and aggressiveness. For this purpose, there are available tools to robustly detect specific p53 isoforms either at RNA- (i.e., with reliable nested qPCR approaches) or protein- [using pantropic antibodies able to recognize a) the whole panel of p53 isoforms or b) specific isoforms according to the domains at N- or C-termini] level. Notably, several of these newly developed antibodies are also good in IHC staining, making them valuable for clinical straightforward analyses.

Overall, we consider the pattern of p53 isoforms’ expression a relevant field of interest in the cancer research panorama, and we believe the study of their functions and characteristics is fundamental in cancer biology. Given that some p53 isoforms present features common to oncogenes and are over-expressed in cancer tissues, they might represent interesting clinical targets for pharmacological inhibition.

## Figures and Tables

**Table 1:** Biological activities of p53 isoforms across different cancer types. The functional impact of each isoform is context-dependent and strictly related to the tumor histotype and cellular environment.

Isoform	Splice Variant	Tumor	Key Effects	References
FLp53	FLp53α	Some	Canonical tumor suppressive functions on apoptosis and cell cycle	[149]
	FLp53β	Some	Senescence induction	[107]
		Renal cell carcinoma	Apoptosis enhancement, good prognosis marker	[109]
		Melanoma	Good prognosis marker	[111]
		Ovarian cancer (p53^wt^)	Poor prognosis marker	[112]
		Gastric cancers (p53^mut^)	Inhibition of cancer cell proliferation	[115]
	FLp53γ	Breast cancer (p53^mut^)	Better prognosis than p53 wt patients	[114]
		Uterine serous carcinoma	Poor prognosis marker	[116]
		Acute myeloid leukemia	Along with high expression of FLp53β, response to chemotherapy is increased	[150]
Δ40p53	All variants	Breast cancer	High Δ40p53:FLp53 ratio promotes cell stemness and correlates with poor prognosis	[127,128]
		Melanoma	Elevated in cancer cells, reduced therapy responses, increased apoptosis	[40,151]
		Lung, colon cancer and melanoma	Induction of netrin-1 pathway addiction for cell survival	[132]
	Δ40p53α	Ovarian cancer (p53^wt^)	Good prognosis marker	[134]
		Glioblastoma	Promotion of cancer cell proliferation	[129]
Δ133p53	All variants	Glioblastoma, serous ovarian carcinoma (p53^wt^)	Dominant negative effect on FLp53, correlation with poor prognosis	[135]
		Serous ovarian carcinoma (p53^mut^)	Good prognosis marker	[135]
		Colorectal cancer	Reduced disease-free survival	[138]
		Gastric cancer	Increased inflammation and cancer cell survival	[139]
	Δ133p53β	Prostate, breast and lung cancer	Increased inflammation and brain metastases	[136,137]
		Glioblastoma	Immunosuppression and chemoresistance	[141]
		Cutaneous melanoma	Poor prognosis marker	[131]
Δ160p53	Δ160p53α	Some	Dominant negative effect on FLp53	[148]
		p53^mut^ cancers	Increased cancer cell survival and invasiveness	[76]
		Cutaneous melanoma	Elevated cancer aggressiveness	[110]
		Uveal melanoma	Possible role in therapy resistance and poor prognosis marker	[43]
		High risk multiple myeloma	Good prognosis marker	[119]
Δ246p53	All variants	Some	Possible triggering of senescence and impairment of tumor formation	[36]

## Data Availability

No new data were created or analyzed in this study. Data sharing is not applicable to this article.

## Abbreviations

The following abbreviations are used in this manuscript:

TAD	TransActivation Domain
DBD	DNA Binding Domain
OD	Oligomerization Domain
RE	Responsive Element
ROS	Reactive Oxygen Species
NMD	Nonsense-Mediated Decay
HGSOC	High Grade Serous Ovarian Carcinoma
HNSCC	Head and Neck Squamous Cell Carcinoma
DSB	Double-Strand Break
HR	Homologous Recombination
NHEJ	Non-Homologous End Joining
BER	Base Excision Repair
TLS	TransLeSion DNA Polymerase
RBPs	RNA-binding proteins
UTR	UnTranslated Region
IRES	Internal Ribosomal Entry Sites
ITAFs	IRES TransActivating Factors
IHC	ImmunoHistoChemistry
NSCLC	Non-Small Cell Lung Cancer
SNP	Single Nucleotide Polymorphism
UPS	Unfolded Protein Response
TIS	Translation Initiation Site
CLL	Chronic Lymphocytic Leukemia
